# Clinical Impact of Pancreatic Metastases from Renal Cell Carcinoma: A Multicenter Retrospective Analysis

**DOI:** 10.1371/journal.pone.0151662

**Published:** 2016-04-11

**Authors:** Paolo Grassi, Ludovic Doucet, Palma Giglione, Viktor Grünwald, Bohuslav Melichar, Luca Galli, Ugo De Giorgi, Roberto Sabbatini, Cinzia Ortega, Matteo Santoni, Aristotelis Bamias, Elena Verzoni, Lisa Derosa, Hana Studentova, Monica Pacifici, Jorgelina Coppa, Vincenzo Mazzaferro, Filippo de Braud, Camillo Porta, Bernard Escudier, Giuseppe Procopio

**Affiliations:** 1 Medical Oncology 1, Fondazione IRCCS Istituto Nazionale Tumori, via G. Venezian 1, 20133, Milano, Italy; 2 Gustave Roussy, 114 Rue Edouard Vaillant, 94805 Villejuif, France; 3 Medical Oncology, IRCCS San Matteo University Hospital Foundation, Viale Camillo Golgi, 19, 27100, Pavia, Italy; 4 Clinic for Haematology, Hemostasis, Oncology and Stemcelltransplantation, Medical School, Carl-Neuberg-Strasse 1, 30625, Hannover, Germany; 5 Dept of Oncology, Palacký University Medical School and Teaching Hospital, I.P. Pavlova 6, 775 20, Olomouc, Czech Republic; 6 Medical Oncology 2, A.O.U.P., Istituto Toscano Tumori, via Roma 67, 56126, Pisa, Italy; 7 Dept of Medical Oncology, Istituto Scientifico Romagnolo per lo Studio e la Cura dei Tumori (IRST) IRCCS, via P. Maroncelli 40, 47014, Meldola, Italy; 8 Dept of Oncology and Haematology and Respiratory Disease, University Hospital, Via del Pozzo 71, 41124, Modena, Italy; 9 Medical Oncology 1 - Candiolo Cancer Institute-FPO, IRCCS, Strada Provinciale, 142 km 3,95, 10060, Candiolo, Italy; 10 Medical Oncology, AOU Ospedali Riuniti, Università Politecnica delle Marche, Via Conca, 71, 60126, Ancona, Italy; 11 Dept of Clinical Therapeutics, Alexandra General Hospital, V. Sofias and Lourou 1 11528, Athens, Greece; 12 Medical Statistics, Trial Center, Fondazione IRCCS Istituto Nazionale Tumori, via G. Venezian 1, 20133, Milano, Italy; 13 Gastrointestinal Surgery and Liver Transplantation Unit, Fondazione IRCCS Istituto Nazionale Tumori, via G. Venezian 1, 20133, Milano, Italy; Seoul National University, REPUBLIC OF KOREA

## Abstract

Pancreatic metastases from renal cell carcinoma are uncommon and their prognostic significance is not well defined. In this analysis we evaluated the outcome of patients with pancreatic metastases treated with either targeted therapies or local treatment to the pancreas. Patients with pancreatic metastases from renal cell carcinoma treated between 1993 and 2014 were identified from 11 European centers. Clinical records were retrospectively reviewed. Kaplan-Meier method and log-rank test were used to evaluate progression-free survival and overall survival. Cox’s proportional hazard models were used for survival analysis. In total, 276 PM patients were evaluated, including 77 (28%) patients treated by either surgery or radiotherapy to the pancreas, and 256 (93%) who received systemic therapy. Median time from nephrectomy to diagnosis of pancreatic metastases was 91 months (IQR 54–142). Disease control rate after first-line TTs was 84%, with a median progression-free survival of 12 months (95% CI 10–14). Median overall survival was 73 months (95% CI 61–86) with a 5-year OS of 58%. Median OS of patients treated with local treatment was 106 months (95% CI 78–204) with a 5-year overall survival of 75%. On multivariable analysis, nephrectomy (HR 5.31; 95%CI 2.36–11.92; *p*<0.0001), Memorial Sloan Kettering/International Metastatic RCC Database Consortium prognostic score (HR 1.45, 95% CI 0.94–2.23 for intermediate vs good vs risk; HR 2.76 95%, CI 1.43–5.35 for poor vs good risk *p* = 0.0099) and pancreatic local treatment (HR 0.48; 95%CI 0.30–0.78 *p* = 0.0029) were associated with overall survival. Difference in median OS between patients with PM and that reported in a matched-control group of mRCC patients with extrapancreatic metastases was statistically significant (*p* < .0001). Pancreatic metastases from renal cell carcinoma usually occur years after nephrectomy, are associated with an indolent behavior and a prolonged survival. Targeted therapies and locoregional approaches are active and achieve high disease control rate.

## Introduction

RCC is the most common kidney cancer in adults [1]. Approximately 20–30% of patients with RCC have metastases at presentation, and 30–50% will eventually develop metastatic disease after nephrectomy [2]. The most common sites of metastases from RCC include lung, lymph nodes, liver, bone and brain. Although PM are rare, with an incidence between 2–11% [3], RCC represents the most common primary tumor leading to PM [4]. PM typically occur a long time after nephrectomy and have been associated with a more favourable outcome [5–7]. Nevertheless, the prognostic role of PM in patients receiving systemic treatment has not been clarified. A retrospective analysis reported a longer overall survival in PM patients treated with TTs [8]. Surgical resection of PM seems to confer a survival benefit [9] but surgery cannot always be performed due to comorbidities or widespread disease.

The landscape of treatment for mRCC has dramatically changed with the introduction of TTs directed against the VEGF and mTOR pathways [10–16]. Several prognostic models have been proposed and validated to predict OS in mRCC patients including MSKCC and IMDC criteria [17,18]. Key variables in these models include ECOG PS, time from diagnosis to initiation of therapy, hemoglobin, calcium and LDH levels for MSKCC and corrected calcium, Karnofsky PS, time from diagnosis to start of therapy, haemoglobin, ANC, as well as platelet counts for the IMDC criteria.

These parameters are used to categorize patients into subgroups with good, intermediate and poor risk of recurrence. Recently, the prognostic impact of the site of metastatic disease such as the negative prognostic impact of bone and liver metastases in patients treated with TTs has been proposed [19,20]. Although PM appear associated with better outcomes, specific risk categories have not been described, and no clinical algorithm, nomogram or published risk criteria incorporates this site of disease. Therefore, it is unclear whether the presence of PM is an independent prognostic variable or it is dependent on other prognostic factors. Reliable prognostic models for outcome in patients with mRCC would represent an important tool that could be used to optimize patient selection for specific treatment strategies.

This retrospective multicenter analysis investigates clinical features and survival in a series of consecutive mRCC patients with PM from eleven different European oncology centers, who were treated with either TTs and/or local treatment to the pancreas.

## Materials and Methods

Consecutive patient series treated at eleven European centers between 1993 and 2014 were retrospectively identified from the mRCC databases of each institution based on the presence of pancreatic metastases (data in S2 Table). Main inclusion criteria were a diagnosis of RCC of any histological subtype, the presence of PM and a treatment for metastatic disease including TTs and/or locoregional approach. Patients who received prior cytokines or who were treated with an investigational agent/combination were included, as well as those who did not receive systemic therapy after pancreatic local treatment. The presence of other sites of disease were allowed to study entry. Baseline demographics, clinical features, systemic treatment, prior local treatment for pancreatic metastases, follow-up and survival data were collected by using uniform database templates to ensure consistent data collection. Objective assessment of tumor changes was performed every three months by computed tomography and/or magnetic resonance imaging according to single Insitutions guidelines. The RECIST version 1.0 criteria were used to evaluate tumor response. Institutional review board approval was obtained from coordinating center (Fondazione IRCCS Istituto Nazionale Tumori, Milano, Italy).

### Statistical analysis

The primary outcome was OS, defined as the time from the diagnosis of PM to death for any cause. Secondary outcomes included PFS, defined as time from the initiation of first-line TTs to disease progression or loss to follow-up; OS calculated from the first diagnosis of metastatic disease to death, and OS for TTs (i.e. time from initiation of first-line TTs to death). In patients still alive at the time of the data base lock, censoring was performed at the time of last follow-up. Furthermore, time intervals from nephrectomy to the diagnosis of PM and time from nephrectomy to first diagnosis of metastatic disease, regardless of the site, were evaluated. The OS of patients with PM was compared with that reported in a population of 330 consecutive mRCC patients with extrapancreatic metastases (control group) who received TTs at Fondazione IRCCS Istituto Nazionale Tumori between 2005 and 2012. A comparison between the main clinical variables (age, prior cytokines, number of targeted therapies) and prognostic factors (ECOG PS, MSKCC prognostic score) of these two groups was performed using the chi-square test for categorical variables, the Kruskal-Wallis test for ordinal variables and the Wilcoxon-Mann-Whitney test for continuous variables. OS and PFS were estimated by the Kaplan-Meier method. Potential prognostic factors for OS were investigated by using univariate and multivariate Cox regression models. A p value <0.05 was considered statistically significant. All analyses were performed using SAS 9. 2 software (SAS Institute, Cary, NC, USA).

## Results

Tables 1 and 2 show the characteristics of patients with PM and the control group respectively. Despite the considerable size of the two patients cohorts, no statistically significant difference in terms of age *p* = 0.585, ECOG PS *p* = 0.574, MSKCC score *p* = 0.199, prior cytokines *p* = 0.900 and number of targeted therapies *p* = 0.109 was observed. The lack of statistical association for the main prognostic factors and clinical characteristics between the control group and the group of patients with metastases to the pancreas allowed to make the two groups comparable, not requiring further statistical balancing methods.

**Table 1:** PM Patients characteristics (n = 276).

	n	%
**Gender**		
F	97	35
M	179	65
**ECOG**		
0		
1		
2		
missing		
**Nephrectomy**		
No	13	5
Yes	263	95
**Histology**		
Clear cell	263	95
Non clear cell	13	5
**Fuhrman grade**		
1	14	6
2	134	57
3	80	34
4	6	3
missing	42	
**MSKCC score**		
Good	96	39
Intermediate	**131**	**54**
Poor	**17**	**7**
missing	**32**	
**Heng prognostic score**		
Good	95	44
Intermediate	112	52
Poor	8	4
missing	61	
**Pancreatic metastases**		
Synchronous	80	29
metacronous	196	71
**Isolated metastatses (pancreas only metastatic site)**	42	15
**Sites of concomitant Metastases**		
Lung	129	47
Lymphnode	76	28
Liver	63	23
Bone	36	13
Kidney (contralateral)	48	18
Adrenal	34	12
Brain	17	6
Thyroid	13	5
Soft tissue	19	7
Other*	47	17
**Pancreatic local treatment**		
Yes	77	28
No	199	72
**Type of local treatment**		
Surgery	68	89
Radiotherapy	6	8
Radiosurgery	2	3
missing	1	
**Number of targeted therapies**		
0	12	5
1	108	42
≥2	135	53
missing	21	
**Cytokines**		
Yes	99	37
No	168	63
missing	9	

*Other: parotid gland, skin, ovaries, spleen, peritoneum, breast, bladder, gallbladder, pleura, heart, colon, eye, testis, ureter, stomach

**Table 2:** Patient characteristics in the control group (n = 330).

	n	%
**Gender**		
F	86	26
M	244	74
**ECOG**		
0	183	56
1	129	39
2	18	5
**Nephrectomy**		
No	42	13
Yes	288	87
**Histology**		
Clear cell	286	87
Non clear cell	44	13
**Fuhrman grade**		
1	15	5
2	98	30
3	127	39
4	47	14
missing	43	
**MSKCC score**		
Good	104	32
Intermediate	157	48
Poor	65	19
missing	3	
**Sites of Metastases**		
Lung	220	3
Lymph node	125	20
Liver	62	10
Bone	94	15
Thyroid	5	1
Other*	115	19
**Number of targeted therapies**		
1	164	49
≥2	166	51
**Cytokines**		
Yes	82	25
No	248	75

*Other: adrenal, soft tissue, pleural, contralateral kidney

In total, 276 patients with PM were evaluated. PM were synchronous to the diagnosis of the primary in 80 cases (29%). The majority of patients (95%) had undergone nephrectomy and 99 patients (37%) received prior cytokines as first systemic treatment. Seventy-seven (28%) patients were treated with pancreatic local treatment including surgery (89%). Forty-two patients (15%) had isolated PM.

Median time from nephrectomy to PM diagnosis was 91 months (interquartile range, IQR, 54–142 months) and the time from nephrectomy to first diagnosis of metastatic disease was 66 months (31–122). Best response to first-line TTs was complete response in 11 cases (5%), partial response in 97 cases (40%) and stable disease in 94 cases (39%), with an overall disease control rate (DCR) of 84%.

**Fig 1:**
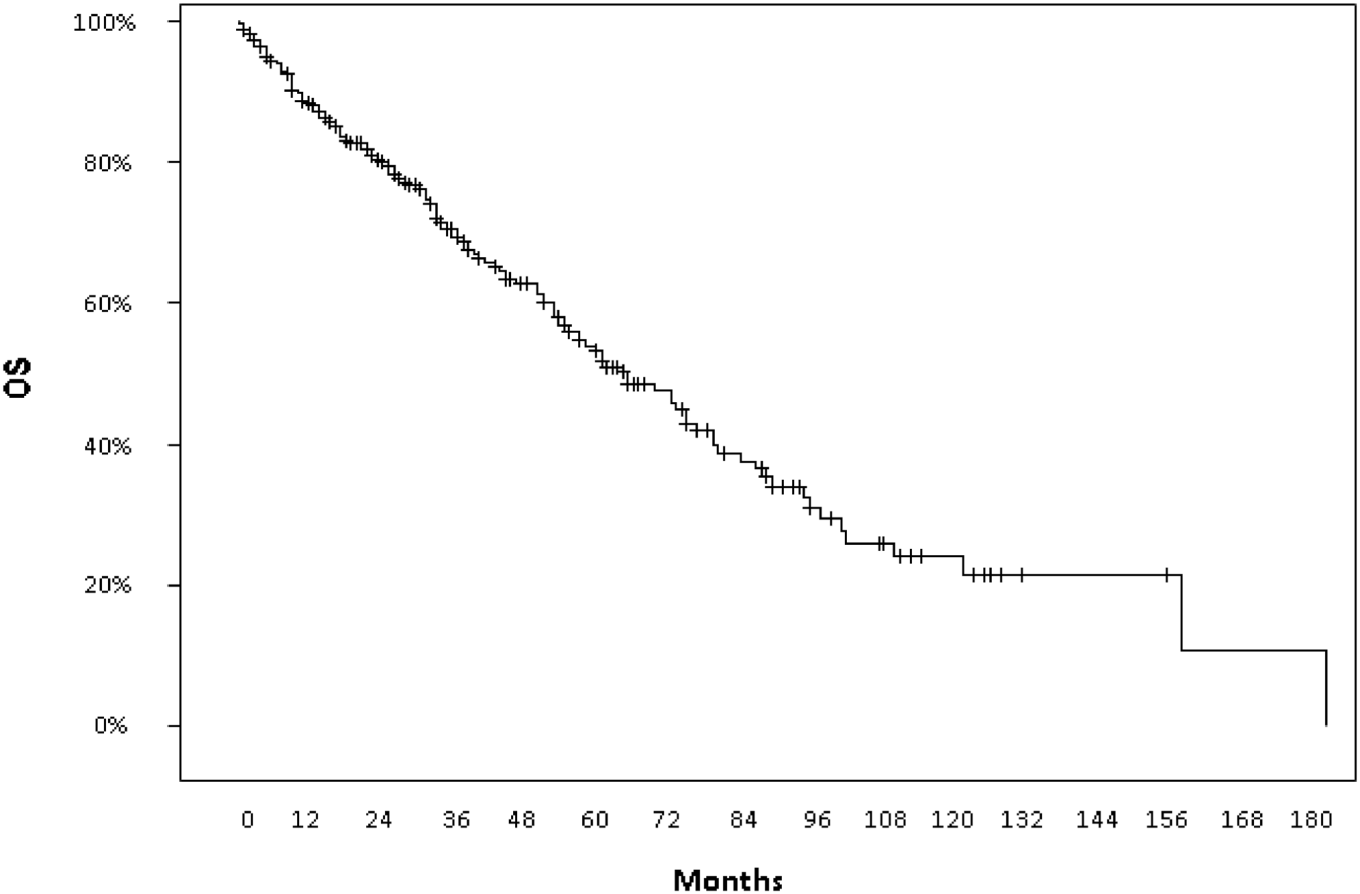
Overall survival calculated from PM manifestation to death.

After a median follow-up of 36 months (95% CI 20–69 months) from the appearance of metastatic disease, median PFS to first-line treatment was 12 months (95% CI 10–14) and median OS calculated from PM diagnosis was 73 months (95% CI 61–86) with a 5-year OS of 58.1% (Fig 1 Overall survival calculated from PM manifestation to death) while median OS calculated from diagnosis of first metastasis to death was 95 months (95% CI 79–112) with a 5-year OS rate of 69.1%.

**Fig 2:**
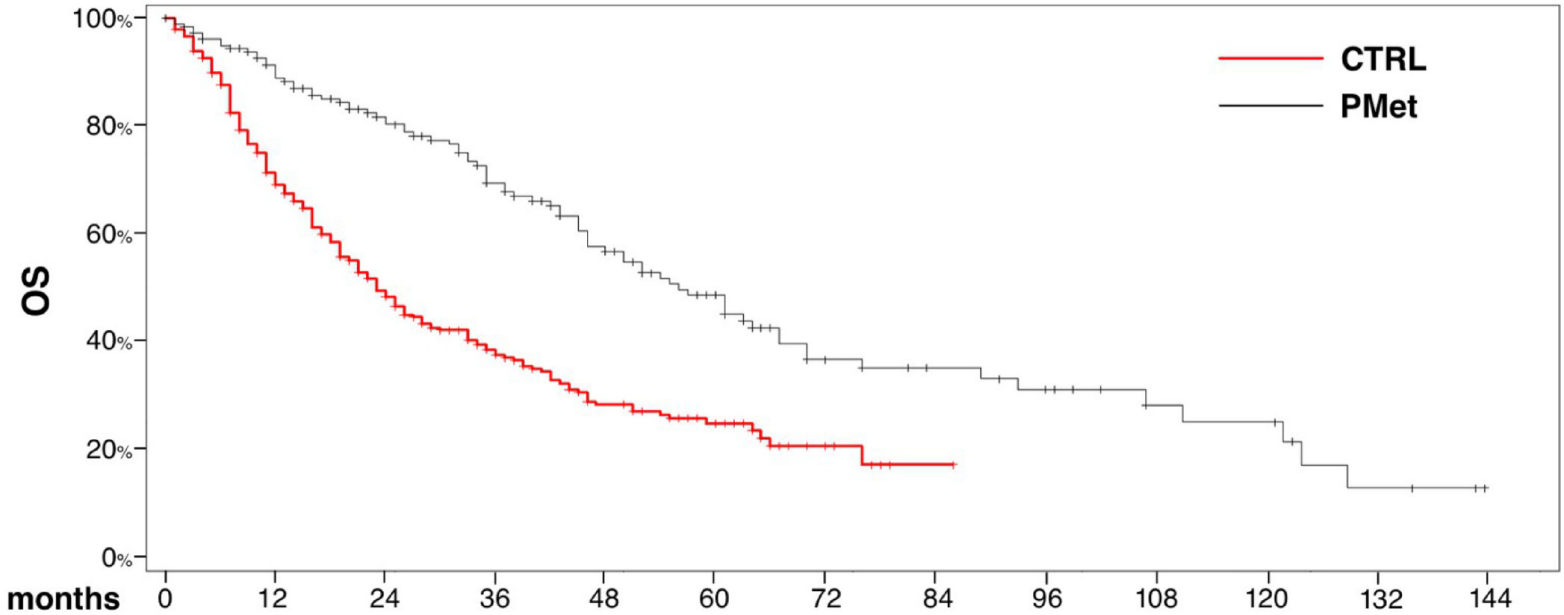
Overall survival calculated from initiation of first-line TTs for PM patients and the control group.

Median OS from the initiation of first-line TTs was 56 months (95% CI 46–67) for PM patients (n = 199) and 23 months (95% CI 20–28) in the control group (n = 330) with a 5-year OS rate of 48.7% and 24.9% respectively (Fig 2 Overall survival calculated from initiation of first-line TTs for PM patients and the control group). This difference was statistically significant (*p* < .0001).

**Fig 3:**
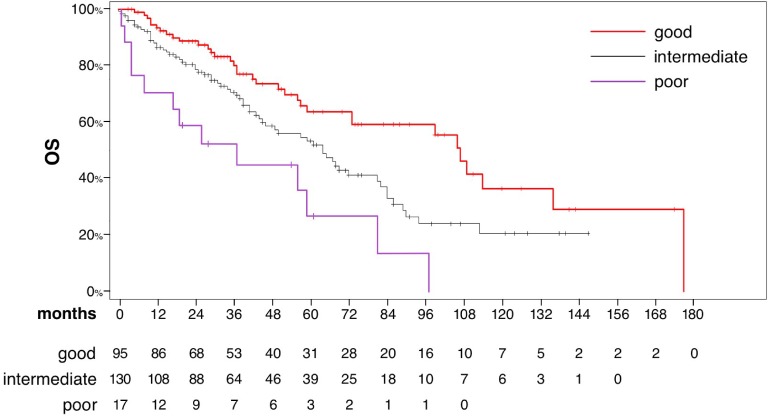
Overall survival in the entire study population according to Memorial Sloan Kettering Cancer Center prognostic risk score.

Median survival according to the MSKCC risk group was 107 months (95% CI 73–136), 64 months (95% CI 45–81) and 37 months (95% CI 4–81) in patients with favorable, intermediate and poor risk, respectively (Fig 3 Overall survival in the entire study population according to Memorial Sloan Kettering Cancer Center prognostic risk score).

**Fig 4:**
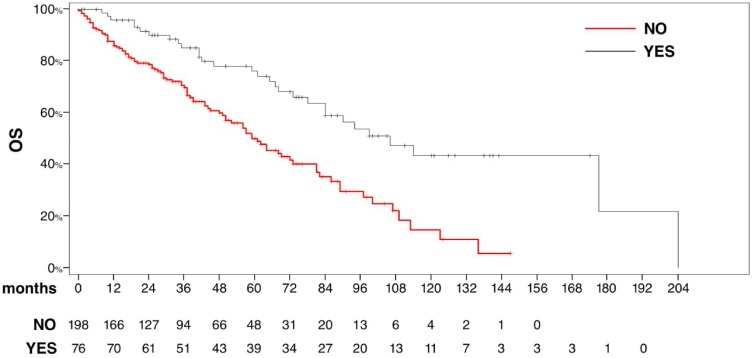
Overall survival for patients who underwent pancreatic local treatment (surgery or radiation therapy) and in those who received TTs only.

Median OS for patients who underwent pancreatic local treatment was 106 months (95% CI 78–204) with a 5-year OS rate of 75% compared with 59 months (95% CI 50–73) in patients who did not (*p* < .0001) (Fig 4 Overall survival for patients who underwent pancreatic local treatment (surgery or radiation therapy) and in those who received TTs only). No differences in terms of MSKCC prognostic group were disclosed between patients who received only TTs and those undergoing pancreatic local treatment (Table 3).

**Table 3:** Outcome according to MSKCC prognostic group for patients who received only TTs and for those who received pancreatic local treatment (surgery or local radiation therapy).

MSKCC prognostic group	n	%	Median OS (months)	95% CI (range)(months)	5-year OS (%)	*p*
**Patients receiving TTs**						0.255
Good	63	36	46	(37–107)	34.7	
Intermediate	97	55	55	(45–70)	49.4	
Poor	16	9	61	(2–70)	51.4	
**Patients who received pancreatic local treatment**						0.377
Good	33	51	83	(65-NA)	88.2	
Intermediate	31	48	106	(29–171)	66.1	
Poor	1	1	-	-	-	

Within the entire patient cohort, three different subgroups were distinguished. These consisted of patients having synchronous PM, patients with PM only who received locoregional treatment and never relapsed, and patients who developed PM after first-line treatment for metastatic disease, respectively. Among 80 (29%) patients with synchronous PM, 2 and ≥3 TTs were administered in 28 (35%) and 22 (27%) cases, respectively. The median PFS to first-line TTs was 10 months (95% CI 7–12) and median OS was 61 months (95% CI 33–120) with a 5-year OS of 51.3%. Twenty-five/42 patients diagnosed with isolated PM received locoregional treatment and 17 of these patients never relapsed. Seventy-seven patients (28%) who developed PM after first-line TTs had a median OS from PM of 67 months (95% CI 35–84) with a 5-year OS of 53.9%.

The univariable Cox proportional regression analysis identified three prognostic factors: MSKCC/IMDC prognostic score (intermediate vs good: HR 1.74, 95% CI 1.74–1.14; poor vs good: HR 3.56 95% CI 1.86–6.81; p = 0.0004), nephrectomy (HR 4.44, 95% CI 2.04–9.68; p = 0.0002) and pancreatic local treatment (HR 0.42 95% CI 0.27–0.65; p<0.0001). On multivariable analysis MSKCC/IMDC prognostic score (intermediate vs good HR 1.45, 95% CI 0.94–2.23, poor vs good HR 2.76, 95% CI 1.43–5.35; p = 0.0099), nephrectomy (HR 5.31, 95% CI 2.36–11.92; p<0.0001) and pancreatic local treatment (HR 0.48, 95% CI 0.30–0.78; p = 0.0029) were confirmed as independent prognostic factors. These results were not modified when the subgroup of patients rendered disease-free after local treatment to the pancreas was excluded (Table 4).

**Table 4:** Univariable and multivariable Cox regression analyses of predictors of overall survival in all PM patients and excluding the subgroup of those patients undergoing local treatment who never relapsed.

**Overall PM patients**				
	**Univariable**		**Multivariable**	
	**HR (95% CI)**	***p* value**	**HR (95% CI)**	***p* value**
**Nephrectomy**				
No vs Yes	4.44 (2.04–9.68)	0.0002	5.31 (2.36–11.92)	<0.0001
**MSKCC/IMDC score**				
intermediate vs good	1.74 (1.74–7.14)	0.0004	1.45 (0.94–2.23)	0.0099
poor vs good			2.76 (1.43–5.35)	
**Pancreatic local treatment**				
Yes vs No	0.42 (0.27–0.65)	<0.0001	0.48 (0.30–0.78)	0.0029
**PM patients undergoing local treatment**				
	**Univariable**		**Multivariable**	
	**HR (95% CI)**	***p* value**	**HR (95% CI)**	***p* value**
**Nephrectomy**				
No vs Yes	4.23 (1.94–9.23)	0.0003	5.31 (2.36–11.93)	<0.0001
**MSKCC/IMDC score**				
intermediate vs good	1.67 (1.09–2.56)	0.0010	1.44 (0.93–2.22)	0.0114
poor vs good	3.33 (1.74–6.40)		2.73 (1.41–5.28)	
**Pancreatic local treatment**				
Yes vs No	0.47 (0.30–0.73)	0.0009	0.52 (0.32–0.85)	0.0092

## Discussion

This multicentric retrospective analysis is the largest cohort of patients reported with this rare presentation of metastatic disease treated in the era of TTs. Overall, PM are associated with more favorable prognostic features, long response to TTs and prolonged survival.

RCC is a heterogeneous disease with different histological subtypes, genetic features and clinical outcomes [21]. RCC often recurs many years after initial nephrectomy and pancreas can be an isolated site of disease relapse associated with a more favorable outcome compared with other metastatic sites [9,22,23] 

Of interest, in the present series we observed a high number of patients with favorable prognostic features such as clear cell histology (95%), prior nephrectomy (95%), metachronous metastases (71%) and late-relapsing disease. In addition, metastatic sites usually associated with poor outcome such as brain and bone were observed in only 6% and 13% of patients respectively [20,24]. Moreover 15% of patients had metastases confined to the pancreas. No relevant differences in terms of baseline chraracteristics between patients who received local treatment to the pancreas and those who received only systemic treatment were observed. Although the median PFS after first-line TTs was comparable with that reported in clinical trials, the median OS appears to be almost twice longer compared to that observed in patients treated within either clinical trials and clinical practice [25]. We observed a median time to presentation of PM after primary tumor resection of 91 months, in line with other reports, and an impressive median OS reaching 95 months when calculated from diagnosis of metastatic disease to death and 73 months when calculated from diagnosis of PM to death, with a 5-year OS rate of 69% and 58% respectively. Difference in OS from initiation of first-line TTs between PM patients and the control group was statistically significant. These findings support the association of PM with an indolent course of disease and are consistent with evidence that the presence of PM has a favorable impact on outcome [8,26]. MSKCC risk group was associated with OS. Other factors such as the presence of concomitant extrapancreatic metastases and ECOG PS were not associated with outcome. On the other hand, the prognostic significance of MSKCC risk group classification in the present cohort seems to be driven by an association with local therapy to the pancreas in the favorable risk group while in patients treated with TTs the differences between MSKCC subgroups were eliminated. The median OS (>5 years) in the poor prognosis group (7%) indicates that MSKCC classification may not be relevant in this population. Noteworthy, we observed an association of PM with other independent favorable prognostic factors, which can account for the more favorable clinical course of this disease. Although in a previous study we reported that PM are an independent prognostic factor [8], the favorable prognostic role of PM remains uncertain. Whether this prognosis is the cause or the consequence of some other favorable factors remains to be determined. A possible explanation could be the long time interval from nephrectomy to the diagnosis of PM, suggesting an extended period of dormancy of cancer cells after radical nephrectomy. This hypothesis may justify the current practice of active surveillance as initial approach for patients with very limited disease, aimed at avoiding treatment-related toxicity and delaying the initiation of systemic treatment whenever radiological and/or symptomatic progression is detected. Moreover, molecular interactions between cancer cells and the pancreatic microenvironment might influence the probability that the cells will grow there (“seed and soil hypothesis”) [27]. As a result, the tumor cells that have affinity to certain metastatic sites might harbor differences in phenotype that lead to peculiar patterns of growth and spread. Zerbi *et al*. documented favorable disease control and survival rates in patients who underwent surgery compared with nonsurgical patients [28]. Tanis *et al*, reported that the 2- and 5-year OS rates were 80.6% and 72.6%, respectively, in patients undergoing surgery, whereas they were 41% and 14% in patients who did not undergo surgery [29]. On the other hand, Santoni *et al* suggested a survival advantage for mRCC patients undergoing pancreatic surgery, although significance was not reached probably due to the low number of patients [27]. Despite to the Tanis et al experience and similarly to the Kalra et al study [30] we reported clinical data from PM patients who were mostly treated with TTs.

Given the heterogeneity of our study population, we evaluated the clinical outcome of three different subgroups of patients with synchronous, metachronous and pancreatic-only metastases respectively. In the subgroup of patients presenting with metastases confined to the pancreas the presence of cases undergoing locoregional treatment who never relapsed may be a confounding factor. After excluding this small subgroup of patients, both univariable and multivariable analyses were not significantly affected, and the association of nephrectomy, locoregional treatment and MSKCC/IMDC score with OS was confirmed. Both subgroups of patients presenting with synchronous or metachronous PM showed an impressive median OS evaluated from first-line TTs of 61 and 66 months respectively with a 5 years OS of more than 50%, further supporting the indolent biological behavior of PM.

In the present series, median OS was almost doubled in patients who underwent pancreatic local treatment, mostly surgery. Most patients undergoing local treatment had metastatic disease confined to the pancreas and therefore seemed the ideal candidates for pancreatic surgery leading to potential survival benefit [3,29]. Although the survival benefit of surgery (or radiotherapy) for metastatic lesions to the pancreas could be confirmed only in a prospective study, a randomized trial comparing surgery with conservative management would be difficult to conduct given the rarity of this presentation and excellent long-term survival after surgery in patients with otherwise lethal disease. Thus, retrospective series still remain the only source of information on patients with these uncommon metastases. The findings of the present study may serve as a basis for diversification of therapeutic strategy in patients with PM.

Our data further confirm the efficacy of TTs in terms of OS, PFS and DCR. Response rate was assessed in 223 patients undergoing first-line TTs, with an encouraging PFS. Treatment strategy is long-term in these patients and at the time of treatment selection should pay special attention to individual chronic toxicity profiles.

Only few patients developed disease progression as best response, regardless of the TTs used. Specifically progressive disease at the initial evaluation was reported only in 9% of first-line cases, while the median PFS was 12 months. These data could be partly explained by the efficacy of TTs and a less aggressive disease. Although this response rate is encouraging, it should be interpreted with caution due to the retrospective nature of the study and lack of comparator arm.

Other limitations of our study include the lack of central pathology or radiology review, and the heterogeneity of patients, who were also treated with different TTs in first-, second- or subsequent line settings.

## Conclusions

PM usually occur many years after nephrectomy, are associated with an indolent disease course and long-term survival. Surgery in oligometastatic disease could improve outcome. TTs are active in this population of patients, resulting in a high DCR. Further prospective studies investigating the biological features of PM appear warranted.

## Supporting Information

S1 Table Datapoints for Figs 1–4. (XLS)

S2 Table Minimal dataset of mRCC patients with PM. (CSV)
